# Performance Evaluation of Large Language Models in Multilingual Medical Multiple-Choice Questions: Mixed Methods Study

**DOI:** 10.2196/81399

**Published:** 2026-03-05

**Authors:** Livia Maria Strasser, Wilma Anschuetz, Fabio Dennstädt, Janna Hastings

**Affiliations:** 1 Medical Knowledge and Decision Support, School of Medicine, University of St.Gallen, St.Jakobstrasse 21, St.Gallen, 9000, Switzerland, +41 71 224 32 00; 2 Department for Assessment and Evaluation, Institute for Medical Education, University of Bern, Bern, Switzerland; 3 Department of Radiation Oncology, Inselspital, Bern University Hospital and University of Bern, Bern, Switzerland; 4 Institute for Implementation Science in Health Care, University of Zurich, Zurich, Switzerland; 5 Idiap Research Institute, Martigny, Switzerland

**Keywords:** medical question answering, LLM, LLM evaluation, education, natural language processing, multiple-choice questions, large language model

## Abstract

**Background:**

Artificial intelligence continues to transform health care, offering promising applications in clinical practice and medical education. While large language models (LLMs), as a form of generative artificial intelligence, have shown potential to match or surpass medical students in licensing examinations, their performance varies across languages. Recent studies highlight the complex influence and interdependency of factors such as language and model type on LLMs’ accuracy; yet, cross-language comparisons remain underexplored.

**Objective:**

This study evaluates the performance of LLMs in answering medical multiple-choice questions quantitatively and qualitatively across 3 languages (German, French, and Italian), aiming to uncover model capabilities in a multilingual medical education context.

**Methods:**

For this mixed methods study, 114 publicly accessible multiple-choice questions in German, French, and Italian from an online self-assessment tool were analyzed. A quantitative performance analysis of several LLMs developed by OpenAI, Meta AI, Anthropic, and DeepSeek was conducted to evaluate their performance on answering the questions in text-only format. For the comparative analysis, a variation of input question language (German, French, and Italian) and prompt language (English vs language-matched) was used. The 2 best-performing LLMs were then prompted to provide answer explanations for incorrectly answered questions. A subsequent qualitative analysis was conducted on these explanations to identify the reasons leading to the incorrect answers.

**Results:**

The performance of LLMs in answering medical multiple-choice questions varied by model and language, showing substantial differences in accuracy (between 64% and 87%). The effect of input question language was significant (*P*<.01) with models performing best on German questions. Across the analyzed LLMs, prompting in English generally led to better performance in comparison to language-matched prompts, but the top-performing models exceptionally showed comparable results for language-matched prompts. Qualitative analysis revealed that answer explanations of the analyzed models (GPT4o and Claude-Sonnet-3.7) showed different reasoning errors. In several explanations, this occurred despite factual accuracy on the represented topic. Furthermore, this analysis revealed 3 questions to be insufficiently precise.

**Conclusions:**

Our results underline the potential of LLMs in answering medical examination questions and highlight the importance of careful consideration of model choice, prompt, and input languages, because of relevant performance variability across these factors. Analysis of answer explanations demonstrates a valuable use case of LLMs for improving examination question quality in medical education, if data security regulations permit their use. Human oversight of language-sensitive or clinically nuanced content remains essential to determine whether incorrect output stems from flaws in the questions themselves or from errors generated by the LLMs. There is a need for ongoing evaluation as well as transparent reporting to ensure reliable integration of LLMs into medical education contexts.

## Introduction

Artificial intelligence (AI) in the form of large language models (LLMs) is rapidly advancing, with multiple applications across the health care sector [1-3]. Despite promising advances, the quality of developed systems remains variable, and extensive evaluation is required for different applications. When adequately implemented, LLMs exhibit substantial potential for supporting clinical practice [4,5]. In the domain of medical decision-making, AI has been shown to perform equivalently to specialists for some tasks [6] and to perform effectively in multiple languages, although performance varies across languages, with English being the predominant training language of most LLMs [7-10].

In the context of medical education, multiple studies have demonstrated that LLMs are able to perform at a level comparable to or even exceeding that of medical students in answering text-based medical licensing multiple-choice questions [11-14]. A comprehensive meta-analysis provided an integrative overview of 45 studies testing the performance of different ChatGPT (OpenAI) models for the task of answering medical licensing examination questions [15]. The findings of this study revealed that the different ChatGPT models exhibited varying levels of performance depending on factors such as the language of the questions (English vs non-English), the difficulty and length of the questions, as well as the type of questions and whether pictures were presented or not. However, the impact of these factors was not consistent between the different models (GPT-4 vs GPT-3.5), and the included studies did not use the same question sets across different languages. Moreover, interpreting model performance is limited by country-specific medical policies, unique local and cultural contexts, and ethical considerations in medical situations [16,17]. A few studies have isolated the effect of language on LLMs’ performance by using comparable examination content across different languages. However, most existing comparisons rely on different national examinations, which vary in structure, limiting the validity of cross-linguistic assessments [18,19]. This study aims to address these gaps by comparing language-dependent performance on identical examination questions of a wide range of LLMs, including open models.

This study investigates how the performance of LLMs varies across languages (German, French, and Italian) in answering medical multiple-choice questions within Switzerland’s unique multilingual context. Using a dataset of example medical licensing examination questions, we explore the performance of a range of different LLMs in answering these questions in German, French, and Italian and how the input and prompt language affects the performance in these different models. In addition, we qualitatively analyze and categorize the types of errors observed in the explanations given by LLMs.

## Methods

### Study Design and Data

We used publicly accessible example question sets on the self-assessment platform from the Institute of Medical Education of the Medical Faculty of Bern, which are provided by the Swiss Federal Office of Public Health to enable medical students to prepare for their examinations [20]. There are 3 question sets, each with 50 questions, resulting in a total of 150 questions. Each question is translated by professional medical translators into the respective examination languages, German, French, and Italian. The provided question sets are comparable in the range of medical knowledge topics and in difficulty level with the federal medical licensing examination in Switzerland. The correct answers to these questions are not publicly accessible. Only when the self-assessment test is submitted does the system provide information about the correctness of the answer given, but not about the answer key. This makes it unlikely that LLMs have been trained with these data. Of the 150 questions, 114 are text-only, while 35 include text and an image. One question included a computed tomography scan video.

Questions containing images or videos were excluded from analysis, as the focus of this study is on multilingual language-processing abilities. Therefore, we restricted the analysis to language-based (text-only) interactions and did not include image-processing or multimodal capabilities. Although recent work has shown substantial performance gains in vision-enabled large multimodal models, incorporating visual items introduces additional variance unrelated to linguistic processing [13,21-24].

The questions were further categorized into subcategories based on medical specialties, purpose of the question, and urgency of the case. Medical specialties were subdivided into general medicine (including internal medicine, neurology, and other smaller subjects, eg, dermatology); surgery and traumatology; pediatrics; gynecology; public health; and other. To differentiate the purpose of the question, questions were categorized into diagnosis (focusing on differential diagnosis, test interpretation, clinical reasoning, physical examination, and diagnostic studies); treatment (addressing therapeutic interventions, medication selection, surgical approaches, and management protocols); and other (public health questions covering prevention, health care systems, and epidemiology). To distinguish urgency within the discussed cases, questions were classified as acute or chronic. Acute conditions were defined as those involving same-day presentations, symptoms lasting less than 1 week, trauma, or infections, whereas chronic conditions were defined as symptoms persisting for more than 1 week, the need for ongoing medication, chronic diseases, or elective scenarios. Furthermore, we categorized the questions based on the response format (single response or multiple responses). Each question was reviewed and categorized for this study by 1 researcher, and categorizations were reviewed and checked by the other researchers. Questions that could potentially fit multiple categories were assigned based on their primary focus or most immediate clinical relevance.

Table 1 shows the distribution of sample multiple-choice questions categorized by specialty, response format, and question modality (text-only, image, and video) across 3 different datasets: text-only (n=114), text and image (n=35), and text and video (n=1).

**Table 1.** Distribution of multiple-choice questions by subcategories, response format, and question modality (text-only, image, and video).

Categories	Text-only (n=114)	Text and image (n=35)	Text and video (n=1)
Specialties, n (%)			
General medicine	72 (63.16)	24 (68.57)	1 (100)
Surgery and traumatology	10 (8.77)	2 (5.71)	0 (0)
Pediatrics	13 (11.40)	6 (17.14)	0 (0)
Gynecology	8 (7.02)	2 (5.71)	0 (0)
Public health and other	10 (8.77)	1 (2.86)	0 (0)
Purpose, n (%)			
Diagnosis	57 (50.00)	25 (71.43)	1 (100)
Treatment	44 (38.60)	8 (22.86)	0 (0)
Other (eg, public health)	12 (10.53)	2 (5.71)	0 (0)
Urgency, n (%)			
Acute	49 (42.98)	20 (57.14)	1 (100)
Chronic	64 (56.14)	15 (42.86)	0 (0)
Response format, n (%)			
Single response	105 (92.20)	32(91.50)	1 (100)
Multiple response	9 (7.80)	3 (8.50)	0 (0)

### Large Language Models

Five different LLMs were applied, as shown in Table 2.

We executed each model 10 times, with the temperature set to the default of 1, top-p to the default of 1, max_tokens set to 10, and no explicit seed set, to estimate the performance in a typical setting where a student is using the technology to support their studying. All source code for the model execution is available from the project’s GitHub repository [25].

**Table 2.** List of LLMs^a^ used in this study.

Model provider	Model name	Implementation
OpenAI	GPT4o	OpenAI API^b^
Meta AI	Llama 3.1-405b-Instruct	DeepInfra API
Meta AI	Llama 3.3-70b-Instruct	DeepInfra API
Anthropic	Claude-Sonnet-3.7‐20250219	Anthropic API
DeepSeek	DeepSeek V3	DeepInfra API

a LLM: large language model.

b API: application programming interface.

### Prompts

In this study, user prompts were structured multilingually. Medical questions were presented to the model in German, French, and Italian while maintaining identical system-level instructions. The LLMs’ responses to these equivalent prompts in different languages of identical examination questions enabled direct cross-lingual performance comparison under controlled conditions.

Two different system and user instruction prompt scenarios were explored: English language system and user instruction prompts while specifying the questions in the different languages in which the examination questions were available (named “English prompts”), and system and user instruction prompts in the same language as the questions were specified (named “language-matched prompts”). This allowed us to contrastively evaluate the impact of designing an interactive medical question answering system based on the target language of the user (language-matched) or the language that the model has the most expertise in (English), with implications for the application of such models in international contexts (Textbox 1).

Textbox 1. **System and user prompts for English and language-matched versions in German, French, and Italian.**

**English**

- System prompt: “*You are a helpful medical question answering assistant. Please carefully follow the exact instructions, return only the answer letter or letters, and do not provide explanations.*”
- User prompt: “*Please answer the following multiple-choice question by selecting the correct response option or options. Question: {}. Response options: A - {}, B - {}, C - {}, D - {}, E - {}. Return only the letter or letters (A, B, C, D, E) corresponding to the correct answer or answers. The correct answer to the question is:*” where the specific question and answer options are inserted from the question dataset.

**German**

- System prompt: “*Du bist ein hilfreicher Assistent bei der Beantwortung medizinischer Fragen. Befolge sorgfältig die genauen Anweisungen, gib nur den/die Antwortbuchstaben an und füge keine Erklärungen hinzu.*”
- User prompt: “*Bitte beantworte die folgende Multiple-Choice-Frage, indem du die richtige/n Antwortoption/en auswählst. Frage: {}. Antwortoptionen: A - {}, B - {}, C - {}, D - {}, E - {}. Gib nur den/die Buchstaben (A, B, C, D, E) an, der/die der/den richtigen Antwort/en entspricht/entsprechen. Die richtige Antwort auf die Frage lautet:*”

**French**

- System prompt: “*Tu es un assistant utile qui répond à des questions médicales. Suis attentivement les instructions exactes, renvoie uniquement la ou les lettre/s de réponse et ne donne aucune explication.*”
- User prompt: “*Réponds à la question à choix multiples suivante en sélectionnant la ou les réponse/s correcte/s. Question: {}. Options de réponse: A - {}, B - {}, C - {}, D - {}, E - {}. Ne renvoie que la ou les lettre/s (A,B,C,D,E) correspondant/es à la/les réponse/s correcte/s. La réponse correcte à la question est:*”

**Italian**

- System prompt: “*Sei un assistente utile per rispondere a domande mediche. Segui attentamente le istruzioni esatte, riporta solo la lettera o le lettere di risposta e non aggiungere spiegazioni.*”
- User prompt: “*Rispondi alla seguente domanda a scelta multipla selezionando la o le opzioni di risposta corrette. Domanda: {}. Opzioni di risposta: A - {}, B - {}, C - {}, D - {}, E - {}. Riporta solo la lettera o le lettere (A, B, C, D, E) corrispondente/i alla risposta corretta o alle risposte corrette. La risposta corretta alla domanda è:*”

### Quantitative Analysis of Model Performance

Model responses were scored in binary as correct or incorrect. For multiple-response items, an all-or-nothing scoring approach was applied: a response was considered correct only if all correct options were selected and no incorrect options were included; ordering of answer options was not considered relevant.

All quantitative data analyses were performed in Python (version 3.12; Python Software Foundation) within a Jupyter notebook running on a MacBook Pro M2 with 32 GB of RAM, while LLMs were executed via online application programming interfaces (APIs). Notable libraries used in the analysis are the Transformers library for language model execution (version 4.45.2; Hugging Face), the OpenAI API for cloud-based model execution (version 1.14.0), the DeepInfra API for executed hosted open models (version 1, via DeepInfra’s OpenAI API), the Anthropic API for executing Anthropic models (version 0.42.0), and the pandas (version 2.2.1; pandas Development Team), and seaborn (version 0.13.2; seaborn Development Team) libraries for data analysis and visualization, respectively. We analyzed the data using generalized estimating equations, as implemented in the statsmodels.genmod library (version 0.14.4; statsmodels Development Team), with a logistic link function and exchangeable correlation structure, to account for the clustering of repeated observations within items. We fit separate generalized estimating equation models to examine the effects of categorical variables, including model, language, and prompt language, on accuracy, each accounting for clustering within items. We fitted separate models due to convergence considerations. However, the fully crossed design ensures effects are not confounded. Effect sizes are reported as odds ratios (OR) with 95% CIs. Differences were deemed significant at a *P* value of <.05. Benjamini-Hochberg correction was applied to control the false discovery rate in subgroup analyses. All source code for the quantitative data analysis is available from the project’s GitHub repository [25].

### Qualitative Analysis of Reasons for Errors

For the qualitative evaluation, the 2 best-performing LLMs from the quantitative analysis, OpenAI’s GPT4o and Anthropic’s Claude-Sonnet-3.7, were selected. Questions answered incorrectly during this quantitative analysis were reentered via the web interfaces (OpenAI WebUI for GPT4o on January 14, 2025; Anthropic WebUI for Claude Sonnet-3.7 on March 17, 2025), each in a new chat session within a single browser (Google Chrome) to minimize potential bias from prior interactions. Questions that were again answered incorrectly during this process were subsequently analyzed qualitatively by a panel of 3 reviewers, 2 medical doctors (WA and FD) and 1 final-year medical student (LMS).

As the quantitative analysis only extracted answers and not explanations, the prompts were revised to explicitly require the LLMs to justify their answer choices, with the goal of gaining deeper insights into the reasons for the observed errors. The questions were entered in German, once with the matched-language prompt in German and once with the English prompt. To better mirror realistic conditions, the model was not given a system prompt. This study aimed to reflect how medical students ordinarily interact with such tools, entering only the multiple-choice question itself with a brief prompt rather than using a form of prompt engineering (Textbox 2).

Textbox 2. **Modified prompts for qualitative analysis in English and German.**

- English prompt: “*Please answer the following multiple-choice question by selecting the correct response option or options. Question: {}. Response options: A - {}, B - {}, C - {}, D - {}, E - {}. Return only the letter or letters (A, B, C, D, E) corresponding to the correct answer or answers and give an explanation to the solution.*”
- German prompt: “*Bitte beantworte die folgende Multiple-Choice-Frage, indem du die richtige/n Antwortoption/en auswählst. Frage: {}. Antwortoptionen: A - {}, B - {}, C - {}, D - {}, E - {}. Gib den/die Buchstaben (A, B, C, D, E) an, der/die der/den richtigen Antwort/en entspricht/entsprechen und gib eine Erklärung zur Lösung.*”

The qualitative analysis was conducted in German or English, depending on the output language of the respective model. The study group consisted of 3 native German speakers and 1 native English speaker. Their respective second and third languages are English, German, and French. The reviewers evaluated and coded all questions with 4 sets of LLM-generated explanations (2 models and 2 prompt languages). For this publication, all presented questions and LLM explanations were translated from German into English with an online translation tool (DeepL; DeepL SE) and professionally proofread.

**Figure 1.**
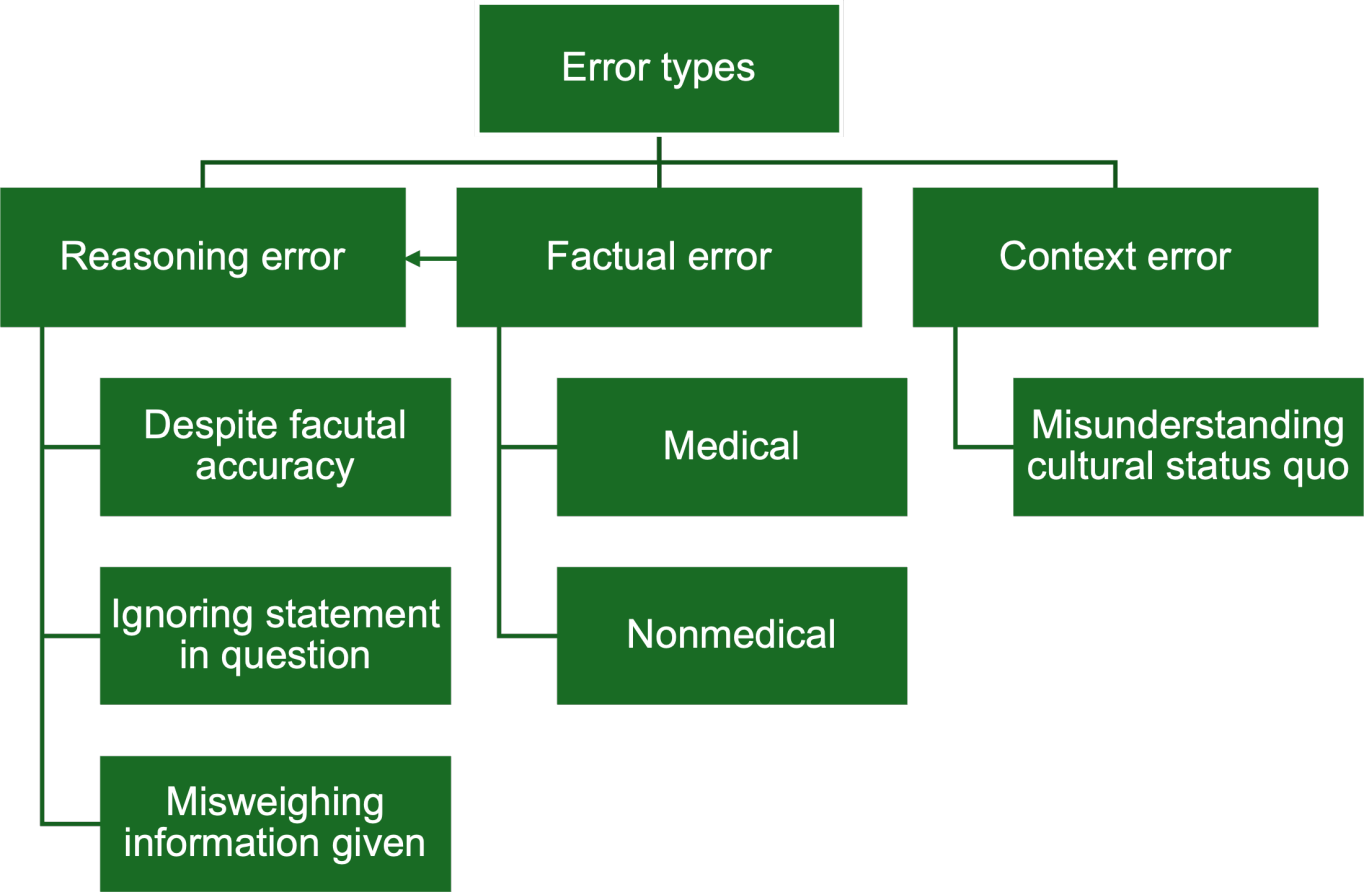
Error typology framework categorizes errors into 3 main types and their subtypes: reasoning errors (despite factual accuracy, ignoring statement in question, and misweighing information given), factual errors (medical and nonmedical), and context errors (misunderstanding cultural status quo).

The qualitative evaluation of the LLMs was conducted using the Framework Method, as described by Gale et al [26]. In recent years, various frameworks have been developed to examine the reasoning processes of physicians and LLMs when responding to medical questions. Our framework was developed with reference to existing studies that have used similar methodologies [27-30]. While other frameworks may differ in structure or focus, our approach was specifically designed to identify the underlying causes of incorrect answers within the explanations given by the respective LLM. The framework was developed through an iterative review process in which multiple reviewers individually examined the LLM explanations and derived preliminary categories and subcategories. These were then discussed collectively, refined through consensus, and cross-validated in subsequent rounds of reanalysis of the LLM explanations. It enabled the investigation of both the basis of the errors and patterns leading to inaccuracies. The framework designed during this process with the distinct error typology is shown in Figure 1. Three main error domains were categorized: reasoning, factual, and context. Reasoning errors involve logical missteps such as ignoring information within the question, reaching incorrect conclusions despite factual accuracy of underlying context information, or misweighing the given information, when complex information is given but the LLM fails to identify the relevant aspects. Factual errors are split into medical and nonmedical, depending on the domain of the incorrect fact. Context errors stem from misunderstandings of the cultural or social background in the context given, such as misinterpreting the cultural status quo or processes in inpatient and outpatient settings or public health matters in Switzerland. This classification aids in systematically identifying and addressing different sources of error. Examples of error types are shown in Textbox 3.

Textbox 3. **Selection of multiple-choice questions and corresponding responses generated by large language models in the English prompt scenario. Each example shows the original question translated into English, the answer options, and the ground-truth label (in brackets) and the qualitative analysis derived from the reviewers’ assessments. For each question, the explanations produced by the large language models are presented (some shortened for readability).**

**Example question 1 reflecting “Reasoning error: ignoring statement in question”:**

“*A Rh-negative, 34-year-old woman in the 16th week of pregnancy is diagnosed with an anti-D titre of 1:128 (positive from 1:16). In her previous first marriage, after two spontaneous abortions, she gave birth to a child at term who required two exchange transfusions due to hyperbilirubinemia. Which measure is indicated? (A) Termination of pregnancy, (B) Doppler of the fetal cerebral artery, (C) Clarification of the Rh blood group status of the father of the child, (D) Administration of anti-D, (E) n/a*” *(Ground Truth: C)*

**Explanation of large language model (GPT4o) in English prompt scenario:**

“*Answer: B, Explanation: An anti-D titre of 1:128 in a Rh-negative pregnant woman indicates significant sensitisation to the Rh-D antigen. (...) The best measure to assess the severity of possible fetal anaemiaanemia is Doppler ultrasound of the fetal arteria cerebri media (A.cerebri media). (...) Why the other options are not correct:*”

“*A: This is not justified at this stage and with this diagnosis. C: This information is no longer relevant in this case, as sensitisation is already present. D: Anti-D prophylaxis is ineffective in the case of existing sensitisation. It is only used to prevent initial sensitisation. Conclusion: The correct answer is B: Doppler of the fetal cerebral artery to monitor for fetal anaemia.”*

**Qualitative analysis of error type made:**

Reasoning error ignoring the statement in question, that the previous child was from another relationship, therefore indicating that this child has another biological father and could be Rh-negative.

**Example question 2 reflecting “Reasoning error: despite factual accuracy”:**

“*A 12-year-old boy cuts the palmar side of the proximal phalanx of his index finger while cutting a branch with a pocketknife. The surgeon consulted found that the sensitivity of the fingertip was intact. Although active flexion of the proximal phalanx is possible, the patient is unable to flex either the seconds or third phalanx. This is a severing of the (A) tendons of the interosseous muscles, (B) tendons of the lumbrical muscles, (C) superficial flexor tendon, (D) deep flexor tendon, (E) superficial and deep flexor tendon*” *(Ground Truth: E)*

**Explanation of large language model (Claude-Sonnet-3.7) in English prompt scenario:**

*“Answer D, The description in the case indicates a severing of the deep flexor tendon (flexor digitorum profundus*). *Analysis of the clinical findings: Cut on the palmar side of the proximal phalanx of the index finger, intact sensitivity of the fingertip (nerve function preserved), active flexion of the proximal phalanx possible, inability to flex the seconds* (*middle) and third (end) phalanx. It is important to know the anatomy of the finger flexors: The superficial flexor tendon (...) attaches to the middle phalanx and enables flexion of the middle joint (PIP - proximal interphalangeal joint), the deep flexor tendon (flexor digitorum profundus) attaches to the distal phalanx and enables flexion of the distal interphalangeal joint (DIP*).

*In the event of an injury at the level of the proximal phalanx, the deep flexor tendon can be severed while the superficial flexor tendon may still be intact. Since the patient cannot flex either the seconds or third phalanx, the function of both the superficial and deep flexor tendon must be impaired. However, the description indicates that active flexion is still possible in the metacarpophalangeal joint (MCP), which is mediated by the lumbrical and interosseous muscles*.

*Answer D (deep flexor tendon) is correct, as an isolated transection of the deep flexor tendon would not fully explain the functional deficit described.”*

**Qualitative analysis of error type made:**

The conclusion does not follow logic and is contradictory, as the whole explanation favors the correct answer E. The large language model states the correct solution during explanation of the anatomy of the muscles that perform flexion in the fingers but fails to give the correct ground truth.

**Example question 3 reflecting “Reasoning error: due to misweighing of information”:**

*“A 20-year-old, previously healthy man falls ill with fever a few weeks after travelling to the tropics. His general condition is reduced; clinically there are palpable lymph nodes at several sites and splenomegaly. There is pancytopenia with haemoglobin 100 g/l (norm 140‐170 g/L), leucocytes 2.0 G/l (norm 4.5‐11 G/l) (granulocytes approx. 75 %, lymphocytes approx. 10 %), and thrombocytes of 65 G/l (norm 150-350 G/l). What is the most likely diagnosis?*

*(A) Acute leukaemia, (B) side effect of mefloquine, (C) acute HIV infection, (D) malignant indolent B-cell non-Hodgkin’s lymphoma with bone marrow involvement, (E) Dengue fever” (Ground Truth: C*)

**Explanation of large language model (GPT4o) in English prompt scenario:**

*“Answer: E, Explanation: The clinical picture of the 20-year-old patient with fever, reduced general condition, palpable lymph nodes, splenomegaly and pancytopenia (...) after a trip to the tropics is typical of an infection with the dengue virus. (...) Why the other options do not fit: A (acute leukaemia): Acute leukaemia could cause pancytopenia but does not explain the travel history and typical course after tropical travel B (side effect of mefloquine): Mefloquine (malaria prophylaxis) is more likely to cause neuropsychiatric side effects, but not pancytopenia and splenomegaly. C (acute HIV infection): Acute HIV infection can cause flu-like symptoms, but not typically pancytopenia with splenomegaly to this extent. D (malignant indolent B-cell non-Hodgkin’s lymphoma): This disease usually progresses insidiously and is unlikely in a young, previously healthy man with no other risk factors. Conclusion: The clinical presentation is best suited to dengue fever (E).”*

**Qualitative analysis:**

The “trip to the tropics” and its association to dengue seems to be overemphasized even though clinical symptoms and the dynamic as well as the onset of the disease are not specific to dengue fever.

**Example question 4 reflecting “Medical Factual error”:**

*“A 6-year-old child has hematomas on various parts of the body. Which location/s is/are suspicious of physical abuse? (A) on the shins, (B) on the back of the hand, (C) on the back, (D) behind the ears, (E) n/a” (Ground Truth: B/C/D*)

**Explanation of large language model (GPT4o) in English prompt scenario:**

“*Answer: C, D, Explanation: Hematomas in children are not uncommon, especially on parts of the body that are frequently injured during normal play or falls. However, there are certain areas of the body where bruising (haematomas) are particularly suspicious for physical abuse. Suspicious localisations:*

*C: Hematomas on the back are unusual as this area is rarely injured during falls or everyday activities. Injuries in this area are often associated with violence*.

*D: Bruising behind the ears is also highly suspicious as this area is not usually affected by accidents. Such injuries may indicate blows or pulling to the head*.

*Why the other options are less suspicious: A: Hematomas on the shins are normal in active children and can often be explained by playing or falls. B: Hematomas on the back of the hand can also be caused by everyday activities and are less obvious for maltreatment. E: (….) Conclusion: The hematomas on the back (C) and behind the ears (D) are suspicious for physical abuse in this case.”*

**Qualitative analysis:**

Bruises on the back of the hand are considered suspicious for physical abuse in a 6-year-old child.

**Example question 5 reflecting “Context error: misunderstanding cultural status quo”:**

*“Young parents call your practice in an emergency at 5 pm in the evening. Their 2-year-old son has suddenly lost consciousness while playing, has tilted backward from a sitting position and has his eyes fixed upwards. He then began to twitch rhythmically with all 4 extremities. The parents poured cold water on his face, after which he slowly regained consciousness a few minutes later. The whole episode lasted 3 minutes. Now he is reduced, tired and seems to have a high temperature. The parents have never experienced anything like this with their son. What is the most appropriate next step in management? (A) The boy must be taken to the nearest emergency centre immediately, (B) You ask the parents to take the boy to your consultation the next day for a check-up, (C) You refer the boy to a neurologist for an EEG scan, (D) You ask the parents to come to your consultation with the boy on the same day to check for a fever focus, (E) No further action necessary” (Ground truth: D*)

**Explanation of large language model (Claude-Sonnet-3.7) in English prompt scenario:**

*“Answer: A, The description indicates a febrile convulsion in a 2-year-old child with the following features: Sudden loss of consciousness followed by rhythmic twitching of all extremities, staring of the eyes upward, short duration (3 minutes), high fever, first episode (parents have never experienced anything similar), postictal state (reduced, tired). Although it is probably a simple febrile convulsion, which is not uncommon in children between 6 months and 5 years of age, the child in this situation should be taken to an emergency centre immediately for the following reasons:1. it is the first episode of a seizure, which requires a thorough work-up to rule out other causes such as meningitis, encephalitis or other neurological conditions, 2. the child is in a reduced state following the seizure, which requires immediate assessment, 3. the level of fever and its cause must be clarified, 4. the young age of the child (2 years) increases the risk of more serious causes*.

*The other options are not appropriate:B: too long a delay in an acute condition. C: not urgent enough EEG is not the first measure. D: important but the situation requires a more comprehensive emergency assessment. E: clearly inappropriate for a first seizure.”*

**Qualitative analysis:**

Context error or subject for discussion: In Switzerland, there is the possibility for urgent outpatient consultation at the family doctor to avoid a consultation at the emergency center in comparable situations. This question cannot be answered internationally due to different availabilities of same-day presentations for children. Further, this question might be challenging for licensing examination candidates, whether an outpatient consultation on the same day (option D) or an immediate emergency consultation (option A) is indicated and could also be discussed in terms of quality assurance.

It is important to note that the identification of reasoning errors does not imply that LLMs do or could possess human-like reasoning capabilities. Rather, the framework provides a structured means to analyze the apparent logic, or lack thereof, within the LLMs’ responses, regardless of the process that generated the response.

### Ethical Considerations

The study is out of scope for national ethical approval as it does not collect any data from humans and is based on publicly accessible datasets and models.

## Results

### Model Accuracy Performance at Answering Questions

**Figure 2.**
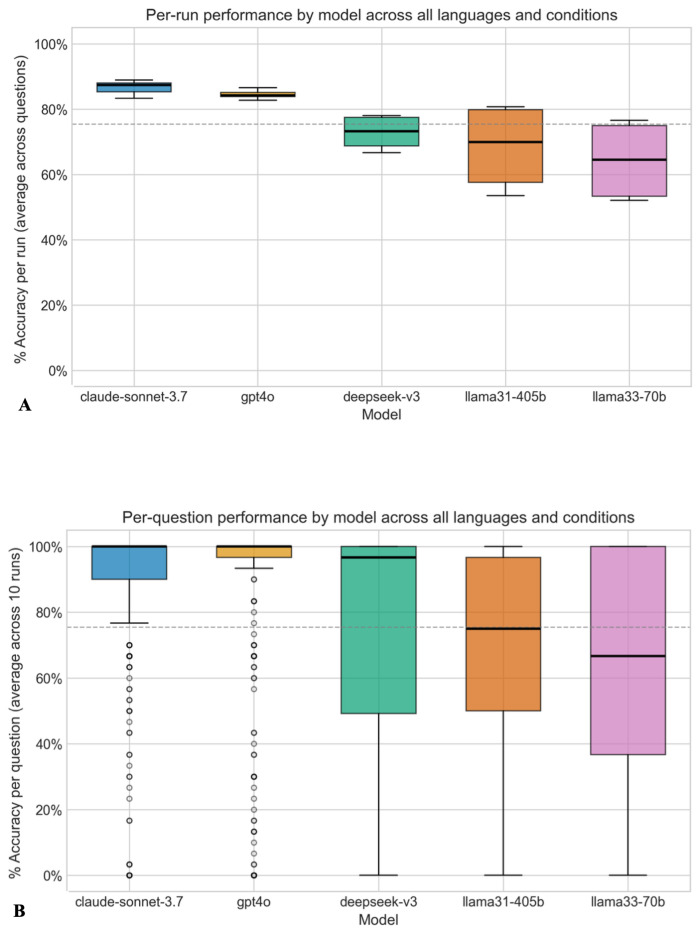
Accuracy of different large language models.

Figure 2 presents the model accuracy results, as averages across the 10 runs per question, across 5 LLMs, for all languages. Claude-Sonnet-3.7 demonstrated the highest mean accuracy (87%, SD 0.34%) and lowest variability, indicating consistent performance. GPT4o also showed strong performance (mean accuracy 84%, SD 0.36%) with slightly more variability. In contrast, Llama3.3-70b, Llama3.1-405b, and DeepSeek V3 exhibited significantly less reliable performance across tasks (mean accuracies of 64%, 69%, and 73%; SDs of 0.48%, 0.46%, and 0.44%; *P*<.001 in all 3 cases). However, all models would be considered to have achieved a passing grade, which can vary in specific threshold but is usually set at around 60% accuracy in written medical licensing examinations.

Figure 2A shows per-run accuracy of different LLMs across all questions and conditions. Accuracy is shown as the percentage of accurate responses per run across questions. Figure 2B shows per-question accuracy of different LLMs across all runs and conditions. Accuracy is shown as % accuracy per question across runs and conditions. A gray dotted line indicates the average across all models. Individual responses per run and table data for the figure are given in Multimedia Appendix 1.

### Performance Variation by Question Language and Prompt Language

**Figure 3.**
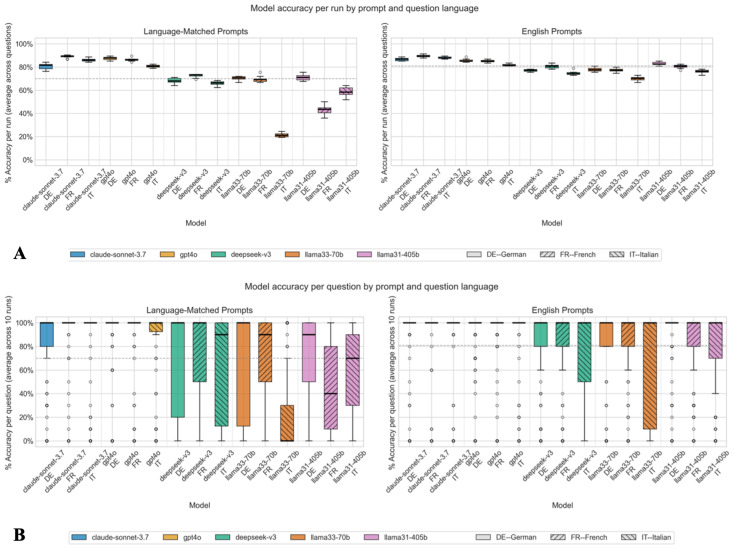
Overall model accuracy across runs and questions by prompt and question language.

Figure 3 illustrates model accuracy across the 3 question languages (German, French, and Italian) and 2 instruction prompt scenarios. As with the overall model comparison, Claude-Sonnet-3.7 consistently achieved the highest accuracy across all languages, with the strongest performance in French and Italian. The lowest performance was achieved in Italian by all other models. Across all models, comparing model performance across the 2 prompt scenarios, English prompts generally led to better or comparable performance relative to language-matched prompts (OR 1.81, 95% CI 1.60-2.05; *P*<.001). Notably, Claude-Sonnet-3.7 and GPT4o showed the highest and most consistent accuracy across all languages and prompt types. Accuracy values were slightly lower for language-matched prompts with Claude-Sonnet-3.7, particularly for German. In contrast, both Llama models exhibited the poorest performance, especially in the language-matched prompt condition for French and Italian.

Figure 3A shows model accuracy per run across all questions, separated by prompt and question language. Accuracy is shown as the percentage of accurate responses per run across questions and conditions. Figure 3B shows model accuracy per question across all runs, separated by prompt and question language (DE=German, FR=French, IT=Italian). Accuracy is shown as % accuracy per question across runs and conditions. The dotted gray line indicates the average across all scenarios. Individual responses per run and table data for the figure are given in Multimedia Appendix 1.

When comparing model performance across the 3 languages, accuracy was highest on average for German-language questions. Therefore, German served as the reference category for the question language statistical comparison. Compared to German, accuracy was not significantly different for French (OR 0.92, 95% CI 0.79-1.08; *P*=.29) but was significantly lower for Italian (OR 0.64, 95% CI 0.54-0.75; *P*<.001).

### Performance Variation by Question Attributes

Regarding the response format, multiple-choice questions with single responses were solved significantly better than those with multiple responses across models (mean accuracy 77%, SD 42% vs mean accuracy 51%, SD 0.50%; OR 0.31; 95% CI 0.12-0.78; *P*<.001). Average accuracies per language, model, and response format (single vs multiple) are illustrated in Figure S1 in Multimedia Appendix 2.

During the quantitative analysis of medical specialties, there were no significant differences in accuracy across clinical question categories (all comparisons not significant after correction for multiple tests). Average accuracies per language, model, and medical specialty are illustrated in Figure S2 in Multimedia Appendix 2.

When looking at the purpose of the question with diagnosis, treatment, and other-related questions as categories, diagnosis questions were used as the reference category. Compared to diagnosis, treatment questions were solved significantly worse (mean accuracy 81%, SD 0.39% vs mean accuracy 70%, SD 0.45%; OR 0.55, 95% CI 0.34-0.91; *P*<.001) while the other-related question also appeared to perform worse than diagnosis, but this difference was not significant (mean accuracy 65%, SD 47% but *P* not significant after correction for multiple tests). Average accuracies per language, model, and purpose category are illustrated in Figure S3 in Multimedia Appendix 2.

There were also no significant differences between questions categorized for urgency, related to acute versus chronic conditions. Average accuracies per language, model, and urgency category are illustrated in Figure S4 in Multimedia Appendix 2.

### Qualitative Analysis

In the qualitative analysis, 12 questions were still solved incorrectly on the web interface with GPT4o and 13 with Claude-Sonnet-3.7, corresponding to those that produced incorrect answers compared to the ground truth in the aggregate quantitative evaluation. Six questions were answered incorrectly by both models. In total, incorrect answers were observed in 20 distinct multiple-choice questions. GPT4o provided incorrect answers to 11 questions when prompted in both German and English, with one additional error occurring exclusively with the English prompt. Similarly, Claude-Sonnet-3.7 gave the same incorrect answers to 8 questions across both languages, while the English prompt resulted in 5 further incorrect responses.

**Figure 4.**
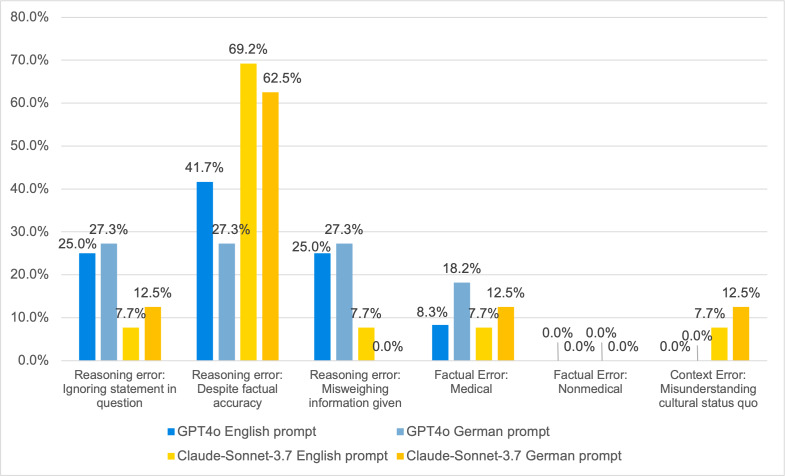
Error analysis of large language models (GPT4o and Claude-Sonnet-3.7) according to the framework with language-matched prompts in German and English language prompt.

Figure 4 shows comparative error analysis and reveals the markedly higher frequency of reasoning errors despite factual accuracy exhibited by Claude-Sonnet-3.7 relative to GPT4o. This error type refers to instances where the model provides information that is factually correct but draws conclusions or inferences that do not appropriately address the questions' requirements. In the English prompt condition, Claude-Sonnet-3.7 produced this type of error in 69% of the erroneous cases (9/13), compared to 42% (5/12) for GPT4o. The pattern persisted in the German prompt condition, with Claude-Sonnet-3.7 showing this error in 62% of the erroneous cases (5/8), while GPT4o’s rate was lower at 27% (3/11).

Textbox 3 presents selected multiple-choice questions, their corresponding LLM-generated answers (from GPT4o or Claude-Sonnet-3.7), and an explanation of the error type and reasoning issues identified. The table focuses on the types and subtypes made by LLMs when responding to the clinical scenarios and includes ground truth answers to the multiple-choice question for comparison.

Additionally, during qualitative analysis, 3 questions’ ground truths were found to be ambiguous given the current state of medical knowledge. One example question is presented in Textbox 4. This example shows the potential risk for LLMs to hallucinate and assume knowledge if asked for an answer, in cases when facts are ambiguous. The model formulated arguments for the answer (C) as ground truth even if the given explanation does not correspond to evidence based on medical knowledge.

Textbox 4. **Example of an ambiguous question and explanation provided by GPT4o.**

**Example question translated into English:**“*A 4-year-old boy with moderate atopic dermatitis is regularly vaccinated according to the Swiss vaccination schedule. Which additional vaccination does he need?*

*(A) Hepatitis A, (B) Hepatitis B, (C) Influenza, (D) Rotavirus, (E) Varicella*” (Ground truth: E)

**Explanation given by large language model (GPT4o) in English prompt scenario:**

“*Answer: C , Explanation: A 4-year-old boy with moderate atopic dermatitis has an increased risk of infection due to the impaired skin barrier and possible inflammatory processes. The influenza vaccination (C) is particularly important as children with atopic diseases are more susceptible to more severe courses of influenza infections. The vaccination not only protects against the disease itself but also reduces the risk of secondary bacterial skin infections, which can occur more frequently with atopic skin. Why the other options are less useful:*

*A: This vaccination is not routinely recommended in Switzerland, but only for certain risk groups or before travellingto endemic areas*.

*B: The hepatitis B vaccination is part of the Swiss vaccination schedule and should already have been carried out. An additional vaccination is therefore not necessary*.

*D: The rotavirus vaccination is recommended in infancy (up to 6 months). This vaccination is no longer indicated at the age of 4 years.*

*E: The varicella vaccination is included in the Swiss vaccination schedule and should have already been given in infancy. An additional vaccination is not necessary if the vaccination schedule has been adhered to*.

*Conclusion: The influenza vaccination (C) is particularly important for a 4-year-old boy with atopic dermatitis, as it protects against possible severe courses and complications.*”

**Qualitative analysis**:

The large language model correctly states that “*The varicella vaccination is included in the Swiss vaccination schedule and should have already been given in infancy.*” It is a correct statement that depicts the disagreement with the ground truth (E) and reflects that the question was developed based on the old Swiss vaccination schedule (prior to 2023). This highlights that the question can no longer be answered as none of the answers would be part of a recommendation. However, the large language model selected answer (C) influenza and provided a rationale which combines the true fact “A 4-year-old boy with moderate atopic dermatitis has an increased risk of infection due to the impaired skin barrier and possible inflammatory processes” with the wrong conclusion that “*The vaccination (….) also reduces the risk of secondary bacterial skin infections*” giving a further example for a reasoning error.

## Discussion

### Principal Findings

This study evaluated the performance of different LLMs in answering multilingual medical licensing examination questions. All models achieved passing-level accuracy, yet performance varied considerably by model, language, and question type. Overall, LLMs performed best on German questions. Prompt language played a key role: while prompting in English generally led to higher accuracy, advanced LLMs such as GPT4o and Claude-Sonnet-3.7 achieved comparable results with language-matched prompts in German and French. Qualitative analysis of answer explanations revealed that both analyzed LLMs (GPT4o and Claude-Sonnet-3.7) exhibited different reasoning error types helping to interpret LLM’s output. The analysis of answer explanations further led to the identification of questions whose content was ambiguous, offering a valuable tool for ensuring question quality.

Our findings are in line with previous findings that LLMs generally exhibit high performance across a variety of medical tasks and languages, reaffirming their potential for supporting multilingual medical education and assessment tasks. However, notable variation exists among models, with some consistently outperforming others in both accuracy and consistency [31,32]. Beyond openly accessible LLMs, state-of-the-art medical models are emerging that demonstrate expert-level performance in real-world clinical tasks and can be deployed locally. However, despite their strong potential, these models are currently better suited for specific applications and ongoing evaluation rather than general clinical use [33]. This highlights the critical importance of model selection and reflection of information presented, as the choice of LLM can substantially influence the quality and reliability of the outputs [34]. Furthermore, the landscape of LLMs is rapidly evolving, with frequent updates and architectural changes leading to substantial shifts in model behavior over time [17]. This dynamic nature poses a challenge for reproducibility and comparability across studies, as the same model may refer to different versions with varying performance profiles [35,36].

Our study showed that prompt language strongly influenced model performance, in line with previous findings [37]. Overall, LLMs tend to be better at following English-language instruction prompts, which we also observed in our study. This highlights persistent limitations in multilingual generalization of LLMs, consistent with LLMs’ predominant training in English, limiting non-English prompt processing and pointing to a need for more robust multilingual training [38]. However, our results show that larger and more advanced models (GPT4o and Claude-Sonnet-3.7) demonstrate strong multilingual question-answering performance. Our analysis indicates that prompt language had a stronger influence on performance than input language, suggesting that the prompt design and model alignment with prompt intent are critical for accuracy and an important skill for medical professionals when using LLMs [39].

The observed performance differences between the smaller models and the large proprietary LLMs have important implications for practical deployment in medical and other data-sensitive environments. In our study, the smaller open-source models consistently produced lower-quality outputs compared with the current closed-source models (eg, GPT4o). Nevertheless, their performance was substantially better when prompted in English, underscoring the need to carefully consider the prompt and context language when such models are used in practice. While locally deployable models offer greater control over data security, they require careful handling of prompt design and a clear understanding of their performance limitations. Thus, our findings highlight the possible trade-off between model performance and data governance requirements [40]. The findings of this study may inform the use of LLMs in other multilingual medical education settings, though factors such as regional terminology, country-specific clinical guidelines, and institution-specific structures may lead to variation.

Moreover, models performed better in diagnostic tasks than in treatment-related questions [41,42], consistent with prior findings [43]. This may be related to a training bias, where diagnostic reasoning is more prevalent in available training data, a pattern that warrants further investigation. The impact of the response format (single vs multiple response) on the accuracy of the answer could be explained by the fact that the multiple-choice questions used to train the models probably consist mainly of single-choice questions as it is the predominant question type in medical education [44]. On the other hand, the exact formulation of the prompts for this question type could also have an impact on the output.

Interestingly, we observed that reasoning errors occurred more frequently when models were prompted in English compared to language-matched prompts, although for the small sample size in our qualitative study, it was not possible to perform statistical tests for significance. A possible explanation could be that, for questions originally written in German, models receiving an English prompt may have internally translated the question into English before generating a response. This intermediate translation step could have introduced subtle semantic inaccuracies, leading to flawed reasoning despite overall factual correctness. Similar translation-related effects have been reported in previous studies, where internal translation processes in multilingual LLMs were shown to increase the likelihood of inconsistencies when prompt and question languages did not align [45]. At the same time, our quantitative results showed that model accuracy per run was comparable in the language-matched scenario in GPT4o and Claude-Sonnet-3.7, suggesting that these advanced LLMs already achieve strong performance in non-English contexts, including German and French. This observation possibly reflects advances in multilingual training data diversity and model alignment, supporting the notion that recent LLMs are becoming more proficient in multilingual reasoning.

As identified in the qualitative analysis, the error type “reasoning error despite factual accuracy” refers to instances where the model presents factually accurate information but draws incorrect or irrelevant conclusions that fail to adequately address the question. These errors are particularly concerning, as their surface-level correctness can obscure deeper reasoning flaws and mislead users [46,47]. In high-stakes domains such as medical decision support or medical education, where correct reasoning is critical, these errors could lead to incorrect conclusions or misleading explanations despite an appearance of accuracy [48]. This finding underscores a key dimension of model reliability that extends beyond factual correctness, highlighting the importance of evaluating reasoning fidelity in addition to factual accuracy when assessing LLM performance [28]. The reasoning error types identified as “ignoring statement in question” and “misweighing information” further demonstrate that although LLMs can process substantial contextual information, they remain vulnerable to subtle pitfalls that may only be apparent to individuals with deep domain expertise. These errors indicate persistent limitations in the models’ ability to appropriately weigh competing pieces of information and to distinguish clinically relevant cues from those that should be considered negligible or misleading. The clinical relevance of these findings extends beyond the experimental setting, as vignette-based questions have the intention to approximate authentic clinical scenarios and to mirror the type of queries that physicians may realistically pose to LLMs in practice. Consequently, the incorrect responses and, in particular, the inaccurate explanations provided by the models are of considerable concern. If such outputs were accepted uncritically, they could mislead clinical decision-making and potentially contribute to diagnostic or therapeutic errors.

The LLMs’ explanation during qualitative analysis helped to recognize changes in medical recommendations or inconsistencies in the question, demonstrating the LLMs’ potential for examination question validation and quality assurance. Professional oversight of the question itself and LLMs’ explanation revealed that the ground truth of the question was based on outdated medical standards. Removal from the examination or thorough revision must be the consequence for such questions. Multiple-choice questions represent an important tool across medical education but are resource-intensive to develop and approve [49]. The implementation of LLMs in question quality evaluation can support flagging ambiguous or poorly formulated questions before they are used in an examination, provided that their use is permitted under local or institutional regulations [50,51]. Future studies should be conducted to further evaluate this potential use case.

### Limitations

Our study has several strengths but also limitations, including response variability of LLMs and the fast-evolving nature of commercially available LLMs, which can affect reproducibility across studies. The performance of the used web-based versions could differ from their API counterparts, which might influence the qualitative findings. The open or commercially accessible LLMs are not intended for medical use but are frequently used by medical students in the respective learning processes due to their seemingly accurate performance [52]. As this study excluded questions with images or videos, the comparison of whole examinations with image-based questions is limited. The qualitative framework is proposed as an exploratory basis for structuring and interpreting the observed outputs and for assessing their potential applicability in other analyses. However, its reliance on human evaluators constitutes a relevant limitation and necessitates further research and validation. Finally, the dataset was derived from a single public Swiss question bank, which may limit the representativeness of topics and linguistic styles. Future studies should include additional data in French, German and Italian to enhance diversity and reduce potential bias.

### Conclusion

In summary, our evaluation confirms that LLMs can handle multilingual medical examination content with high accuracy. However, meaningful differences in performance across models, languages, and clinical tasks underscore the importance of careful model selection, prompt design, and language. Although the overall results favor English prompts, the specific findings from GPT4o and Claude-Sonnet-3.7 indicate that advanced models may perform comparably with language-matched prompts, highlighting the need for further exploration of this effect. In this study, LLM-based qualitative analysis supported the understanding of errors made by LLMs and the identification of ambiguous questions, highlighting its potential for quality assurance in medical assessments. Human oversight of language-sensitive or clinically nuanced content remains essential to determine whether incorrect answers stem from flaws in the question itself or from errors generated by the LLM. Continued evaluation and transparent reporting will be important as models rapidly evolve and become further integrated into educational and assessment settings.

## Supplementary material

10.2196/81399 Multimedia Appendix 1 Raw data to figures shown in the manuscript.

10.2196/81399 Multimedia Appendix 2 Supplementary data with additional figures referenced in the manuscript.

## Abbreviations

AI: artificial intelligence
API: application programming interface
LLM: large language model
OR: odds ratio
