# Supplementary material for: Performance Evaluation of Large Language Models in Multilingual Medical Multiple-Choice Questions: Mixed Methods Study
Source: JMIR Med Educ. 2026 Mar 5;12:e81399. doi: 10.2196/81399 (PMC12978932; doi:10.2196/81399)
Supplement: Multimedia Appendix 2 [file mededu-v12-e81399-s002.docx]

**SUPPLEMENTARY FIGURES**


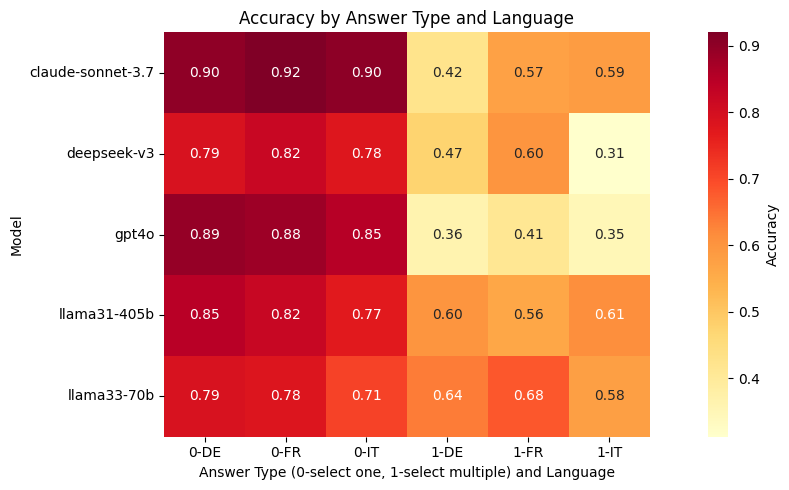


**Figure S1.** Model accuracy across answer type (single choice or multiple-choice) and language impact (DE=German, FR= French, IT= Italian).

**
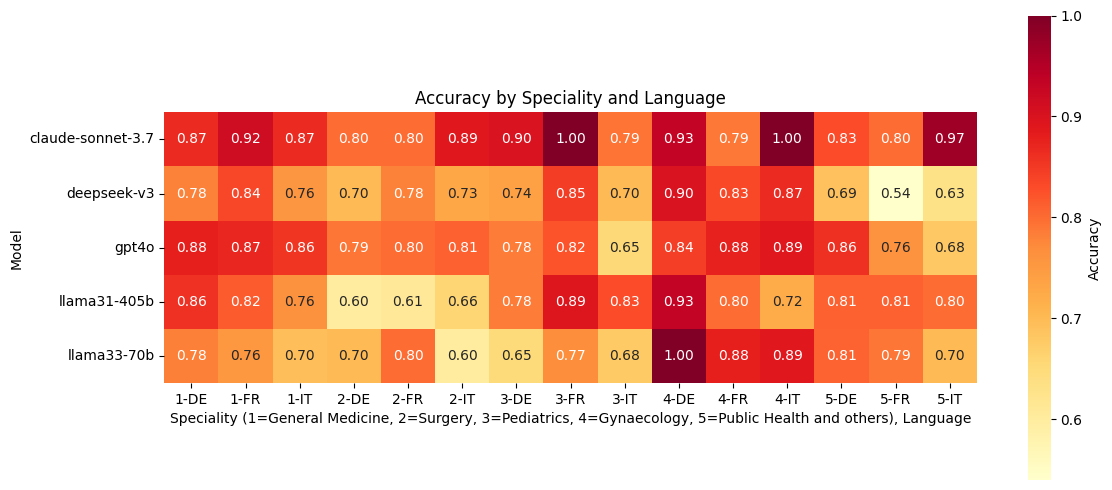
**

**Figure S2.** Model accuracy across different medical specialties (General Medicine (1), Surgery (2), Paediatrics (3), Gynaecology (4), and Public Health & others (5)) and languages (German=DE, French=FR, and Italian=IT). The heatmap colour intensity corresponds to accuracy. Aside from Gynaecology (p-value=.01), the between-group differences were not statistically significant.


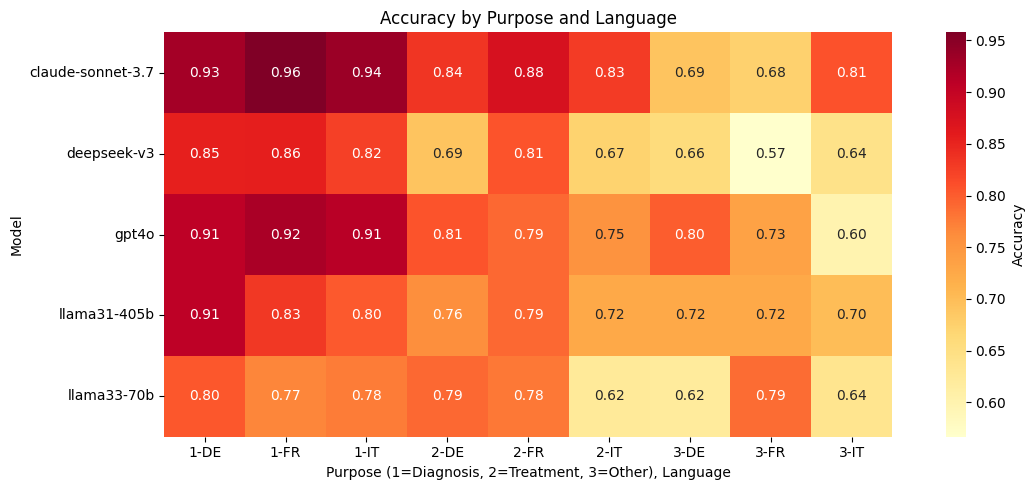


**Figure S3.** Model accuracy across different tapes of purpose (Diagnosis (1), Treatment (2) or other (3)) and language (DE=German, FR= French, IT= Italian).


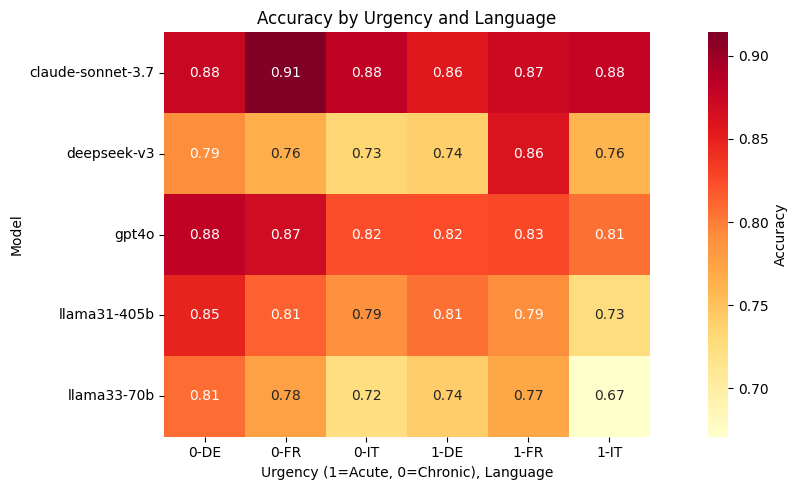


**Figure S4.** Model accuracy across different types of urgency (acute =1 and chronic = 0 problems) and language (DE=German, FR= French, IT= Italian).
